# Development of second generation peptides modulating cellular adiponectin receptor responses

**DOI:** 10.3389/fchem.2014.00093

**Published:** 2014-10-17

**Authors:** Laszlo Otvos, Daniel Knappe, Ralf Hoffmann, Ilona Kovalszky, Julia Olah, Tim D. Hewitson, Roma Stawikowska, Maciej Stawikowski, Predrag Cudic, Feng Lin, John D. Wade, Eva Surmacz, Sandor Lovas

**Affiliations:** ^1^Department of Biology, Temple UniversityPhiladelphia, PA, USA; ^2^Faculty of Chemistry and Mineralogy, Center for Biotechnology and Biomedicine, Institute of Bioanalytical Chemistry, Universität LeipzigLeipzig, Germany; ^3^1st Institute of Pathology and Experimental Cancer Research, Faculty of Medicine, Semmelweis UniversityBudapest, Hungary; ^4^Department of Medicine, The University of MelbourneMelbourne, VIC, Australia; ^5^Torrey Pines Institute for Molecular StudiesPort St. Lucie, Florida, FL, USA; ^6^Florey Institute of Neuroscience and Mental Health and School of Chemistry, The University of MelbourneMelbourne, VIC, Australia; ^7^Sbarro Institute for Cancer Research and Molecular Medicine, Temple UniversityPhiladelphia, PA, USA; ^8^Department of Biomedical Sciences, Creighton UniversityNE, USA

**Keywords:** antiproliferation, biodistribution, dimeric peptide, molecular dynamics simulations, nanomolar activity

## Abstract

The adipose tissue participates in the regulation of energy homeostasis as an important endocrine organ that secretes a number of biologically active adipokines, including adiponectin. Recently we developed and characterized a first-in-class peptide-based adiponectin receptor agonist by using *in vitro* and *in vivo* models of glioblastoma and breast cancer (BC). In the current study, we further explored the effects of peptide ADP355 in additional cellular models and found that ADP355 inhibited chronic myeloid leukemia (CML) cell proliferation and renal myofibroblast differentiation with mid-nanomolar IC_50_ values. According to molecular modeling calculations, ADP355 was remarkably flexible in the global minimum with a turn present in the middle of the peptide. Considering these structural features of ADP355 and the fact that adiponectin normally circulates as multimeric complexes, we developed and tested the activity of a linear branched dimer (ADP399). The dimer exhibited approximately 20-fold improved cellular activity inhibiting K562 CML and MCF-7 cell growth with high pM—low nM relative IC_50_ values. Biodistribution studies suggested superior tissue dissemination of both peptides after subcutaneous administration relative to intraperitoneal inoculation. After screening of a 397-member adiponectin active site library, a novel octapeptide (ADP400) was designed that counteracted 10–1000 nM ADP355- and ADP399-mediated effects on CML and BC cell growth at nanomolar concentrations. ADP400 induced mitogenic effects in MCF-7 BC cells perhaps due to antagonizing endogenous adiponectin actions or acting as an inverse agonist. While the linear dimer agonist ADP399 meets pharmacological criteria of a contemporary peptide drug lead, the peptide showing antagonist activity (ADP400) at similar concentrations will be an important target validation tool to study adiponectin functions.

## Introduction

Adiponectin is a relatively large (244 amino acid) cytokine that is normally produced by the fat tissue and found in human serum at concentrations of 2–20 μg/mL making it one of the most abundant proteins in systemic circulation (Arita et al., 1999; Ryan et al., 2003; Chiarugi and Fiaschi, 2010). Circulating adiponectin levels are inversely correlated with body mass index (BMI) (Galic et al., 2010). Adiponectin is considered a protective hormone exhibiting beneficial effects against insulin resistance, cardiovascular disease, inflammatory conditions, and cancer (Barb et al., 2007; Schaffler et al., 2007; Ziemke and Mantzoros, 2010). The protein circulates in trimeric, hexameric, and higher order complexes although less abundant monomeric forms have been also detected (Fang and Sweeney, 2006). Two adiponectin receptors, AdipoR1 and AdipoR2, have been identified. Both are seven-transmembrane domain proteins containing an N-terminal intracellular portion and the C-terminal extracellular-domain (Yamauchi et al., 2003; Kadowaki and Yamauchi, 2011).

Although some anti-diabetic drugs (e.g., metformin, a biguanide) (Hadad et al., 2008; Gonzalez-Angulo and Meric-Bernstam, 2010) as well as caloric restriction (Jiang et al., 2008) can partially mimic adiponectin action and induce AMPK (5′ adenosine monophosphate-activated protein kinase) signaling in cancer tissues, specific, and selective compounds targeting AdipoR were not available before 2011. Adiponectin protein-based biological modulators are still not available, partly due to difficulties in converting the full size adiponectin protein into a viable systemic regulator. The main reason of the non-druggability of adiponectin protein is the extreme insolubility of the C-terminal domain and larger peptide fragments thereof (Otvos et al., 2011a). Consequently, we attempted to produce small peptides that would produce similar or superior biological effects as adiponectin protein but would be suitable for pharmacological modifications. Adding to the difficulties in experimental design and drug development, AdipoR1 and AdipoR2 also form homomeric complexes that influence their ligand binding properties (Almabuada et al., 2013) and make these proteins poorly soluble chemical entities.

In 2011 we reported on the identification of the adiponectin active site and the design and functional characterization of adiponectin-based peptide compounds for the activation of AdipoR using glioma and breast cancer (BC) model systems (Otvos et al., 2011a). The lead peptide, the 10-mer ADP355, primarily acts through AdipoR1 and induces typical adiponectin signaling pathways in cancer cells (Otvos et al., 2011a). Similar to full-sized adiponectin and its C-terminal globular domain, peptide ADP355 exhibits cell-type specific cytostatic activity on glioma and BC cells. Notably, however, AMPK phosphorylation, believed to be a marker of adipokine function, is observed only for MCF-7 hormone sensitive but not MDA-MB-231 hormone resistant BC cells. The cell-type dependence of AMPK involvement in adiponectin signaling and the differences in the cellular effects of various adiponectin protein preparations were noted earlier (Wijesekara et al., 2010). ADP355 shows no toxic properties to normal mice up to a 10 mg/kg bolus intraperitoneal (ip) dose.

ADP355 contains 4 unnatural amino residues, and due to these modifications the peptide (peptidomimetic) shows remarkable stability in human serum. When administered ip at 0.5–1 mg/kg/day, peptide ADP355 exhibits typical adiponectin effects in rodents, that is stabilization of metabolic functions, and prevention of brain injury caused by HIV protease inhibitors (Pepping et al., 2014) and reduces weight gain and improves biomarker pattern in animals on long-term high-fat diet (Wintrob et al., 2013). A recent study used ADP355 as a reference compound to identify non-peptidic AdipoR agonists from a 10,000-member natural product library, but even the best hit retained μM IC_50_ activities (Sun et al., 2013). Another small molecule library identified an orally available AdipoR agonist called AdipoRon, but the reported low micromolar receptor binding kinetics and mid-micromolar *in vitro* efficacies are even more inferior compared to the earlier reported non-peptide ligands (Okada-Iwabu et al., 2013).

Currently AdipoR antagonists do not exist but could find application in diseases characterized by adiponectin overabundance. For example, serum adiponectin levels are reproducibly higher in severe rheumatoid arthritis patients than in the control group (Ebina et al., 2009). The expression levels of both AdipoR subtypes are also remarkably higher in lesional than in non-lesional areas of osteoarthritis cartilage (Kang et al., 2010). Thus, an AdipoR antagonist would potentially offer a novel treatment option to arthritic diseases. Perhaps more realistically, an AdipoR antagonist would represent a highly-awaited target validation tool for AdipoR agonist drug development.

Although the signaling and growth modifying effects of the full protein is frequently studied in skeletal muscle, cardiovascular, liver, and lung cell lines, the correct evaluation of the readout of these assays are not without controversy (Dadson et al., 2011). First, agents that induce endogenous adiponectin production can have opposite effects compared to adding exogenous adiponectin as was shown on fibrogenesis in hepatic stellate cells (Potter and Mezey, 2007). Second, in mouse primary hepatocytes, adiponectin can exert *in vitro* functions independent of signaling pathways detected *in vivo* (Miller et al., 2011). Third, and most significant to synthetic peptides, proper signaling, and inhibition of smooth muscle cell proliferation by adiponectin requires proteolytic cleavage to shorter forms (Fuerst et al., 2012). Thus, for evaluating new ADP355 peptide derivatives and comparing their cellular functions, we keep using cancer cell models that work satisfactorily and consistently in our hands and proliferation inhibition/enhancement as assay readout.

At a very high dose (10 mM), peptide ADP355 prevents stiffness-associated focal adhesion kinase phosphorylation in myofibroblasts grown on soft surfaces better than 10 μg/mL full-sized adiponectin (Kumar et al., 2014). The first goal of the current studies was to investigate the utility of ADP355 in non-solid neoplasms and fibrotic diseases, and improve the IC_50_ of AdipoR agonists to high picomolar-low nanomolar levels. Recognizing the high demand for an AdipoR antagonist for target validation in biomedical research (and potentially to treat autoimmune diseases), our second goal was to convert the AdipoR agonist ADP355 into an AdipoR antagonist. For this purpose we used the technology that was successful in turning leptin-based ObR agonist peptides into picomolar antagonists (Otvos and Surmacz, 2011). In the current report, we describe the development of second generation AdipoR cellular response modifying peptides (peptidomimetics), with agonist and antagonist activity levels expected of contemporary drug leads.

## Materials and methods

### Peptide synthesis

The peptides listed in Table 1 were assembled on a SYRO2000 (MultiSynTech GmbH, Witten, Germany) multiple synthesizer with standard 9-fluorenylmethoxycarbonyl/*tert*-butyl (Fmoc/*^t^*Bu)-chemistry. ADP355 (peptide 355), Chex-DSer-8 (400), ADP355 linear branched dimer (399), ADP355 cyclic dimer (398), and the trimeric (500) peptides were synthesized on Rink amide 4-methylbenzhydrylamine (MBHA, 0.6 mmole/g; Iris Biotech GmbH, Marktredwitz, Germany) (355, 400), TentaGel R RAM (0.19 mmole/g; Rapp Polymere GmbH, Tuebingen, Germany) (399, 500), and Fmoc-Asp (PEG-PS)-OAll (0.16 mmole/g; PerSeptive Biosystems GmbH, Hamburg, Germany) (398) resins on a 25 μmole scale. Amino acid derivatives (Iris Biotech or Orpegen Pharma GmbH, Heidelberg, Germany) were activated *in situ* with diisopropy-carbodiimide (DIC) in the presence of 1-hydroxybenzotriazole (HOBt) (Knappe et al., 2011). The C-terminal Asp-OAll of the ADP355 cyclic dimer was deprotected using 3 equivalents of tetrakis (triphenylphosphine)palladium (0) catalyst (15.5 g/L, w/v) in a mixture of chloroform, acetic acid, and 4-methylmorpholine (37:2:1, v/v/v) under nitrogen atmosphere for 2 h. The resin was washed twice with (i) *N,N*-diisopropyl-ethylamine (0.5%) in dimethyl-formamide (DMF; Biosolve, Valkenswaard, Netherlands), (ii) sodium diethyl-carbamate in DMF (0.5%), (iii) HOBt in DMF (0.25 M) and (iv) DMF. Cyclization was achieved by activation with 2 equivalents of DIC/HOBt. The DY675 fluorophore (674 nm absorption/688 nm emission) was coupled as pre-formed N-hydroxy succinimide ester to resin-bound ADP355 and the linear dimer. The final peptides were cleaved with trifluoroacetic acid (TFA) containing 12.5% (v/v) of a scavenger mixture (ethane dithiol, m-cresole, thioanisole, and water, 2.5:5:5:5, v/v/v/v) and precipitated with cold diethyl ether after 2 h. The precipitated peptides were washed twice with cold diethyl ether, dried, and purified by reversed-phase high performance liquid chromatography (RP-HPLC) using a linear aqueous acetonitrile gradient in the presence of 0.1% (v/v) TFA as ion pair reagent (Jupiter C18-column, 10 mm internal diameter, 250 mm length, 5 μm particle size, 30 nm pore size; Phenomenex Inc., Torrance, USA). The final products were characterized by RP-HPLC and matrix-assisted laser ionization/desorption time of flight mass spectrometry (MALDI-TOF-MS; 4700 Proteomic analyzer; Applied Biosystems GmbH, Darmstadt, Germany) using α-cyano-4-hydroxycinnamic acid (Bruker Daltonics GmbH, Bremen, Germany; 4 g/L in 60% aqueous acetonitrile containing 0.1% TFA) as matrix.

**Table 1:** **Adiponectin receptor response modifier peptides**.

**Number/Name**	**Sequence^a^**	**Function**	**Monoisotopic mass [M + H]^+^ (calculated/measured)**	**Retention time (min)**
355	H-DAsn-Ile-Pro-Nva-Leu-Tyr-DSer-Phe-Ala-DSer-NH_2_	First generation agonist (published)	1109.59/1109.60	28.5
ADP355	DY675-ADP355		1797.84/1797.87	
398	Cyclo (DAsn-Ile-Pro-Nva-Leu-Tyr-DSer-Phe-Ala-DSer-His-Pro-DAsn-Ile-Pro-Nva-Leu-Tyr-DSer-Phe-Ala-DSer-His-Asp)-OH	Control cyclic peptide to verify structure calculations (new)	2670.33/2670.24	32.8
ADP355 + C1 Cyclic dimer				
399	(H-DAsn-Ile-Pro-Nva-Leu-Tyr-DSer-Phe-Ala-DSer-His-Pro)_2_-Dab-NH_2_ (branched)	Second generation agonist (new)	2769.44/2769.48	30.2
ADP355 + C1				
Linear dimer	DY675-ADP355 + C1		3457.61/3457.67 (single label)	
400	H-Chex-Gly-Leu-Tyr-DSer-Phe-Ala-DSer-NH_2_	First generation antagonist (new)	868.45/868.45	26.6
Chex-DSer-8				
500	(H-DSer-Asn-Ile-Pro-Nva-Leu-Tyr-DSer-Phe-Ala-Tyr-His-Pro)_2_-Dab-Dab(H-DSer-Asn-Ile-Pro-Nva-Leu-Tyr-DSer-Phe-Ala-Tyr-His-Pro)-NH_2_	Control trimeric agonist (new)	4686.41/4686.44	
Branched trimer				

*While the chemical and biological properties of ADP355 have been publisher earlier, the two dimeric agonists, the trimeric peptide and the antagonist are new to this study*.

a *Chex, Dab, Nva, and D denote 1-amino-cyclohexane carboxylic acid, 2,3-diamino butyric acid, norvaline, and D-amino acids, respectively*.

### Serum stability

For serum stability studies (Powell et al., 1993) 10 μL of an aqueous peptide stock solution (3 mg/mL) was added to 390 μL pooled mouse serum in triplicate. The peptide-serum mixtures were thermostated at 37°C. After 0, 30, 120, and 240 min, 95 μL aliquots were mixed with 25 μL trichloroacetic acid (15%, w/v) and incubated for 10 min on ice. The precipitated proteins were separated by centrifugation (5 min, 12,000 × g, Eppendorf MiniSpin). The supernatant (90 μL) was neutralized with 8.5 μL aqueous sodium hydroxide solution (1 M) and mixed with 3% aqueous acetonitrile containing 0.1% TFA (121.5 μL). After centrifugation (5 min, 12,000 × g, Eppendorf MiniSpin) a 100 μL solution was analyzed by RP-HPLC using a Jupiter C18 column (2.0 mm inner diameter, 150 mm length, 5 μm particle size, 30 nm pore size; Phenomenex, Torrance) on a Beckman Gold HPLC System at 60°C. The gradient from 0.1% TFA in water (eluent A) to 60% acetonitrile in 0.1% aqueous TFA (eluent B) was developed using 1% B/min and the amide bond was monitored at 214 nm.

### Cell proliferation

MCF-7 BC and K562 chronic myeloid leukemia (CML) cells were grown in standard RPMI 1640 medium. Seventy-five thousand MCF-7 and 100,000 K-562 cells grown in RPMI 1640 medium containing 10% fetal bovine serum (FBS, for MCF-7) or 0.5% bovine serum albumin (BSA, for K562) were seeded into wells of 24-well culture plates. After 24 h, MCF-7 cells were shifted to K562 medium (serum free). After an additional 24 h, the cultures were treated with various concentrations of peptides or a mixture of peptides for 72 h and counted. The assays were done in triplicates and repeated twice from different cell preparations. The cell numbers were expressed as means ± standard error (SE). Statistical analysis was done with a SlideWrite graphical software package (Encinitas, USA).

### Renal myofibroblast differentiation

Renal interstitial fibroblasts were isolated from fibrotic rat kidneys using explanting methods described previously (Hewitson et al., 2001). Kidney tissue for explants was derived from obstructed rat kidneys 3 days post-unilateral ureteric obstruction. Cultures were propagated by mincing the tissue into pieces, placing them into gelatin- (Sigma, St. Louis, USA) coated Petri-dishes (Nunc, Roskilde, Denmark), and adding DMEM (Dulbecco's modified Eagle's medium, Sigma) supplemented with 10% FBS (Bovogen, Melbourne, Australia) and penicillin/streptomycin antibiotics (MP Biomedicals, Santa Ana, USA). Explants were maintained at 37°C with media being changed twice a week. At confluence, primary outgrowths were trypsinized and subcultured for subsequent experiments. Subcultured cells were characterized cytochemically and by growth characteristics as fibroblasts, a proportion of which were defined as myofibroblast (activated fibroblasts) based on positive staining for α-smooth muscle actin (SMA) (Karamifar et al., 2013).

For quantitative studies, cells were grown on cover slips (Nunc, Roskilde, Denmark) to semi-confluence and exposed to control media or media supplemented with the ADP355 peptide in triplicate. After 48 h cells grown on coverslips were fixed in ice-cold methanol. Following blocking with non-immune serum and incubation with anti-SMA, (Dakopatts, Glostrup, Denmark) cover slips were washed in phosphate buffered saline (PBS), incubated with biotinylated anti-mouse immunoglobulin (Vector, Burlingame, USA), washed in PBS, and incubated with avidin–biotin complex (Vector) and 3,3′-diaminobenzidine (Sigma). Finally, cells were counterstained with Harris haematoxylin (BDH Kilsyth, Australia) and mounted with Aquamount Gurr (BDH). Cells with positive staining were enumerated and expressed as a percentage of total cells counted.

### *In vivo* biodistribution

Twenty μg of DY675-coupled peptides ADP355 (355) and linear dimer (399) were injected ip or subcutaneously (sc) into anesthetized female Skh1 hairless mice. While sleeping, the animals were placed into the fluorescence microscope chamber. One minute-long fluorescence exposure pictures were taken with a Kodak 4000 MM camera set to 700 nm emission wavelength immediately after inoculating the mice, 10 min later and 1 h after drug administration. The mice were photographed both from their abdominal and dorsal sides (Otvos et al., 2011b). All experiments with vertebrate animals were approved by the Animal Health Committee of Semmelweis Medical School.

### Replica exchange molecular dynamics simulations

Conformational properties of adiponectin analogs were studied with the GROMACS 4.5.7 software package (Hess et al., 2008) using REMD simulations (Sugita and Okamoto, 1999). For the simulations the AMBER99SB-ILDN-NMR force field parameters (Li and Bruschweiler, 2010) were used. Atom types and partial atomic charges for norvaline were derived from leucine and isoleucine. Force field parameters for Chex were obtained from Grubisic et al. (2012). Atom types for Dab were derived from lysine and the atomic partial charges were calculated with YASARA AutoSMILES (www.yasara.org/autosmiles). Peptides were solvated with TIP3P waters (Jorgensen et al., 1983) in a truncated dodecahedron, Na^+^ and Cl^−^ ions were added to neutralize the charge of the systems and the final concentration of NaCl was adjusted to 150 mM. Simulations were 150 ns long, integration time step was 2 fs and all bond lengths were constrained using the LINCS (Hess et al., 1997) method. Electrostatic interactions were calculated with the particle mesh Ewald method (Darden et al., 1993) using 1.0 nm cutoff and the van der Waals interactions were switched off between 0.7 and 0.9 nm. Non-bonded pair lists were updated at every 0.02 ps. Simulations were performed in an NPT (constant number of particle, pressure, and temperature) ensemble by coupling the system to a bath of constant 1 bar pressure with 1.0 ps coupling time. The peptides and water plus NaCl molecules were coupled separately to external heath bath with 0.1 ps relaxation time. Temperatures ranged from 290 K to 383 K, using 20% acceptance ratio, the number of replicas were calculated using the http://folding.bmc.uu.se/remd/ web server (Patriksson and Van der Spoel, 2008). Exchanges between neighboring replicas were attempted at every 2 ps.

Convergence of an REMD simulation was determined by calculating the configurational entropy of a peptide chain in a demultiplexed trajectory using the quasi-harmonic approximation (Andricioaei and Karplus, 2001) in the essential dynamics analysis module of GROMACS. Results presented here are from the trajectories at 300 K. During the simulations for peptides 25, 355, and 400 the configurational entropy reached a plateau after 100 ns, therefore, the last 50 ns of these trajectories were used for subsequent analysis. For peptide 399, the configuration entropy reached a plateau for the last 78 ns, therefore, this portion of the trajectory was analyzed. The dihedral principal component analysis (dPCA) method (Mu et al., 2005) was used to explore the free energy landscape of peptides using the first two principal components dPCA1 and dPCA2. The low energy structures on the free energy surfaces were determined by clustering the data with the R 3.0.2 program (R Core Team R, 2013) using the partition around medoid clustering methods with 10 clusters.

## Results

### Screening of ADP355 in diseases involving AdipoR activity

#### Chronic myeloid leukemia

**Figure 1:**
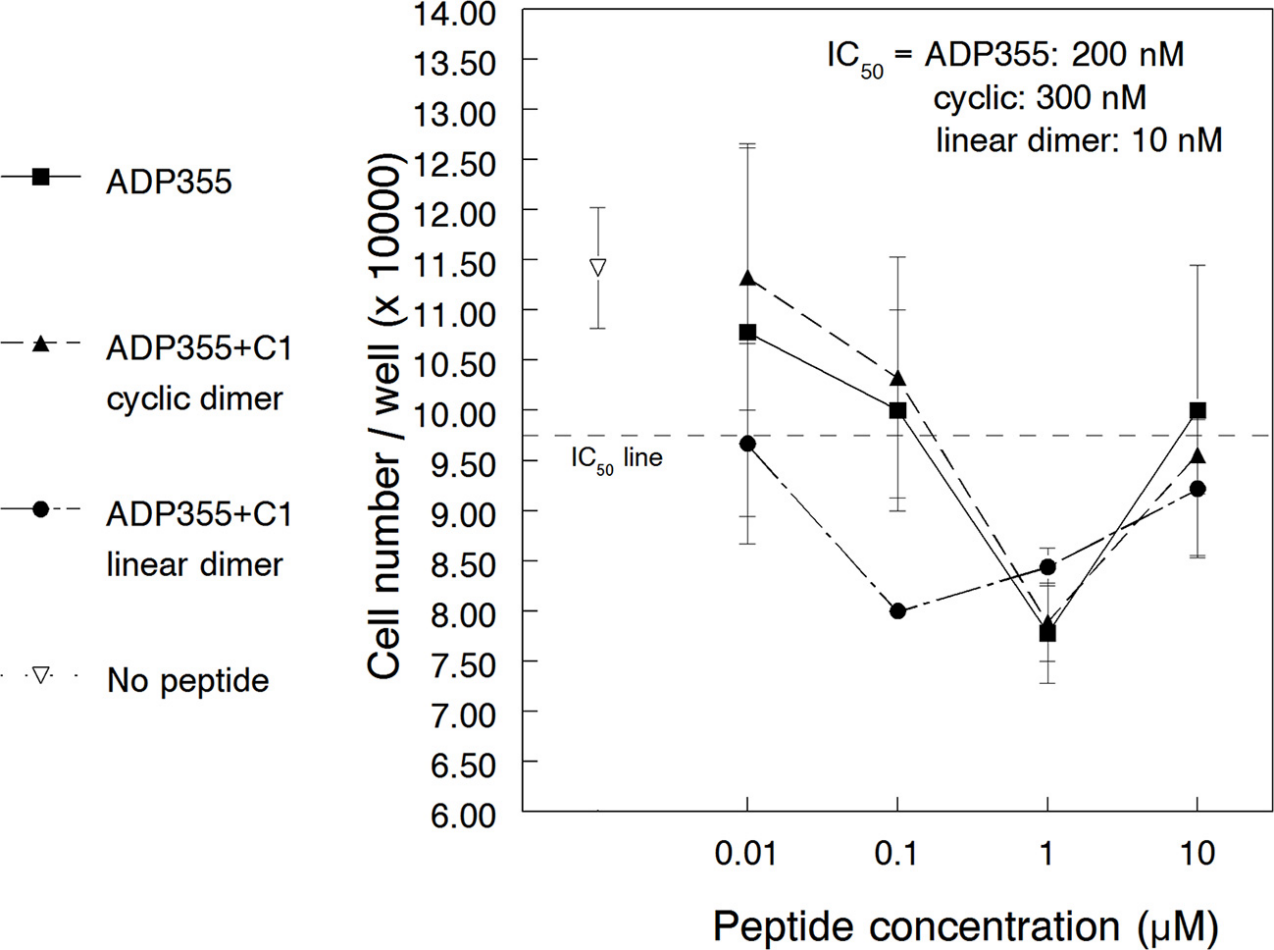
**Effect of adiponectin receptor agonist peptides on adiponectin receptor-positive K562 chronic myeloid leukemia cells *in vitro***. The peptides (355, 398, and 399) were added in 10 nM–10 μM concentration. The empty triangle indicates cell growth without any peptide added. The relative IC_50_ line is drawn halfway between uninhibited cell growth and maximal growth inhibition by the peptides (lowest level of cell growth in the current assay).

To investigate the utility of AdipoR agonists to non-solid neoplasms, we studied the effects of peptide ADP355 (peptide 355, Table 1) on the proliferation of the CML cell line K562. When added at 10 nM–1 μM, ADP355 reduced the 3-day proliferation rate in a dose-dependent manner (Figure 1). Maximum effect was observed at 1 μM that corresponded to 20–40% reduction in surviving cells (across 3 independent assays) and compared favorably to a 20–25% maximum cell death for the MCF-7 BC model in the earlier study (Otvos et al., 2011a). The relative IC_50_ of our first assay was estimated at 200 nM indicating that higher ADP355 concentrations are required to inhibit CML than BC cells where the IC_50_ is 10–50 nM (in hormone responsive and triple negative BC, respectively). At later assays, K562 cells became 10-fold more sensitive to AdipoR agonist treatment either due to stress imposed by low nutrient media conditions, or due to the increased number of cell passages that potentially reflected the increased expression of AdipoR1 during CML pathogenesis (Ozturk et al., 2012). While at later passages 10 nM concentration of ADP355 showed some activity, maximum growth inhibition was consistently detected at 1 μM. As seen earlier for this peptide with glioblastoma and BC cells (Otvos et al., 2011a) and for leptin receptor (ObR) antagonists (Beccari et al., 2013) at high dose (10 μM) the pharmacological effects were reversed, and ADP355 inhibited CML cell growth to a smaller extent (Figure 1). Similar bell-shaped dose-response curves were observed for a non-peptide agonist of the glucagon-like peptide (GLP) 1 receptor (Knudsen et al., 2007) and a peptidomimetic of the granulocyte-colony stimulating factor (Tian et al., 1998). For the GLP-1 receptor agonist the high dose reversal of the pharmacological profile was explained as a non-specific antagonistic effect. Based on analogy with leptin receptor agonists (Kovalszky et al., 2010), such high dose antagonistic effects of our adiponectin peptides cannot be excluded.

#### Myofibroblast differentiation

**Figure 2:**
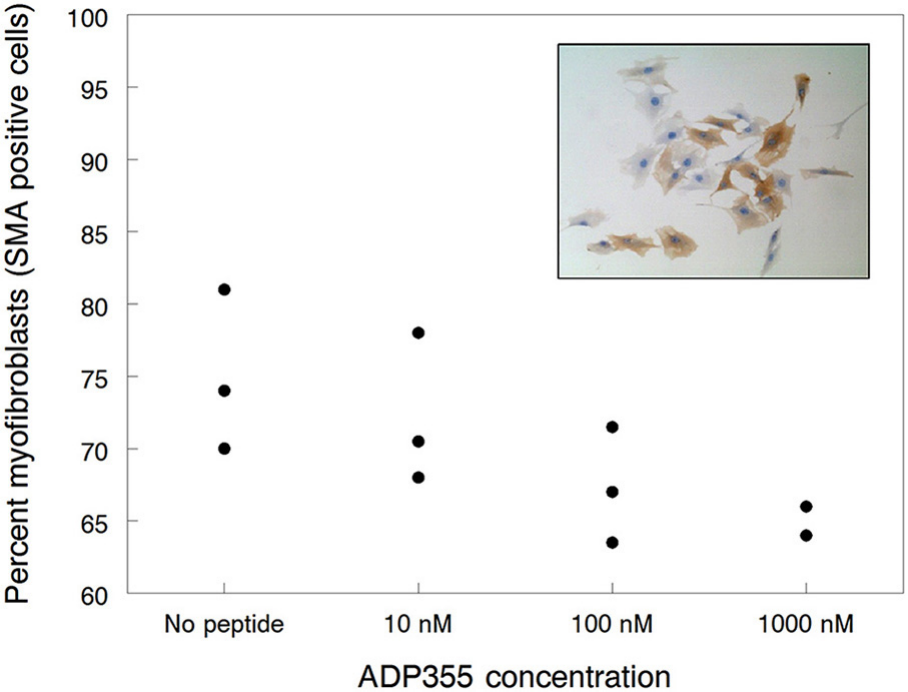
**Inhibition of renal myofibroblast differentiation by adiponectin receptor agonist peptide ADP355**. The filled circles represent individual data points. The inset shows how the effects of 1 μM ADP355 was quantified by measuring the proportion of fibroblast cultures staining brown for α-smooth muscle actin (SMA), a marker of myofibroblast differentiation, and therefore fibroblast activation (compare with Materials and Methods).

First we studied the effects of ADP355 (peptide 355) on kidney fibroblast proliferation. Treatment with 10 nM–1 μM peptide inhibited the growth by 28–43% but no concentration dependence could be observed and the deviation among parallels were relatively high (data not shown). A clearer picture emerged when we studied α-smooth muscle actin (SMA) positive cells, a measure of fibroblast activation (inset to Figure 2). ADP355 inhibited renal myofibroblast differentiation by 4, 11 ± 4, and 14 ± 2% when added at 10, 100 nM, and 1 μM, respectively (Figure 2). While these results do not warrant the continued research of ADP355 in kidney disease, they verify the involvement of AdipoR in tissue fibrosis and suggest that improved AdipoR agonists may find utility in fibrotic diseases.

### Design of an AdipoR antagonist

Based on the success of our first-in-class AdipoR agonist ADP355 and recognizing the need for a similar AdipoR antagonist, we modified the composition of ADP355 to attenuate AdipoR functions and signaling. The starting point of the design was the activity of the 396-member peptide library we made earlier and from which the ADP355 agonist was selected, and a new central hexamer named peptide 3–8 (Table 2). Biological activity screening in MCF-7 cells suggested that the nanomolar agonistic activity can be restricted to the C-terminal octapeptide fragment, and the residues C-terminal of Pro3 bind but do not activate AdipoR. While Pro1 in the C-terminal octamer (Pro3 in ADP355) is replaceable, Gly2, Tyr4, Phe6, Ala7, and DSer8 should be kept for binding. Because Nva3 recovers activation, Leu, the original amino acid in native adiponectin, should be used in this position. DSer5 was added to ADP355 (as DSer7) for increased stability, and as this residue promotes binding but does not support agonist functions, it should be kept in the new AdipoR antagonist. In the new antagonist Pro1 was replaced with 1-amino-cyclohexane carboxylic acid (Chex1), extremely useful as an N-terminal cyclic aliphatic amino acid mimic in designer antibacterial peptides (Cudic et al., 2002). These considerations led to derivative 400 (ADP400), H-Chex-Gly-Leu-Tyr-DSer-Phe-Ala-DSer-NH_2_ (we called it Chex-DSer-8), as a potential AdipoR antagonist (Tables 1, 2).

**Table 2:** **Activity of members of a targeted agonist library that served as a basis of the design of Chex-DSer-8, the first-in-class adiponectin receptor antagonist peptidomimetic**.

**ID**	**Peptide sequence^a^**	**Agonistic activity**	**Structural considerations**
			**For length**
25	Asn-Ile-Pro-Gly-Leu-Tyr-Tyr-Phe-Ala-Tyr	Normal	Antagonist is inside peptide 25 (P25)
26	Pro-Gly-Leu-Tyr-Tyr-Phe-Ala-Tyr-His-Ile	Normal	Inside 3–10 of P25
3–8	Pro-Gly-Leu-Tyr-Tyr-Phe	None	Keep residues 9–10 of P25
			**For antagonist architecture**
24	His-Cys-Asn-Ile-Pro-Gly-Leu-Tyr-Tyr-Phe	Normal	Residues C-term of Pro (until Tyr10 in P25)
156	His-Cys-Asn-Nva-Pro-Gly-Leu-Tyr-Tyr-Phe	None	bind but do not activate
			**Antagonist positional refinements Pro1-Tyr8**
92	Cpc-Gly-Leu-Tyr-Tyr-Phe-Ala-Tyr-His-Nva	Normal	Pro1 is replaceable
157	Asn-Ile-Pro-Nva-Leu-Tyr-Tyr-Phe-Ala-Tyr	Increased	Keep native Gly in position 2
288	His-Cys-Asn-Nva-Pro-Gly-Nva-Tyr-Tyr-Phe	Reduced	Nva in position 3 recovers activation; keep Leu3
290	Pro-Gly-Leu-DSer-Tyr-Phe-Nva-Tyr-His-Ile	None	Keep Tyr4 and Ala7 for binding
223	Asn-Ile-Pro-Gly-Leu-Tyr-DSer-Phe-Ala-Tyr	Weakest among P25 analogs	DSer in position 5 for increased stability binds but does not support agonist function
90	DLys-Cys-Asn-Ile-Pro-Gly-Leu-Tyr-Tyr-Cpc	Reduced	Keep Phe in position 6 for binding
91	DAsn-Ile-Pro-Gly-Leu-Tyr-Gly-Phe-Ala-DSer	Normal	DSer in position 8 retains binding

a *Cpc, Nva, and D denote 1-amino cyclopentane carboxylic acid, norvaline, and D-amino acids, respectively*.

### Screening the biological activity of ADP400, Chex-DSer-8

While obviously we do not intend to use the AdipoR antagonist as an oncogenic agent, for the first screen of general antagonistic properties we investigated the ability of peptide Chex-DSer-8 (peptide 400) to recover the proliferation of different neoplastic cell lines known to be inhibited by the AdipoR agonist ADP355 (peptide 355).

#### Chronic myeloid leukemia model

**Figure 3:**
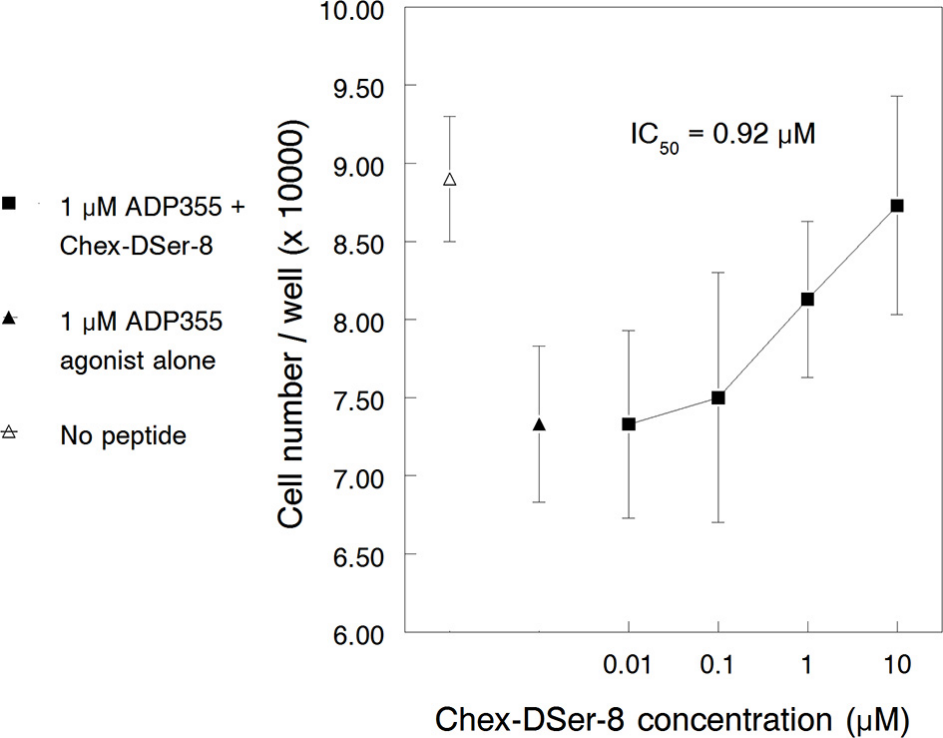
**Peptide Chex-DSer-8 (ADP400) counteracts the K562 cell growth inhibitory effects of peptide ADP355 with a relative IC_50_ of 920 nM**. The empty and filled triangles show the cell growth with no peptide added (uninhibited cell growth) and with 1 μM ADP355 alone (full growth inhibition), respectively.

Similar to the findings in the first paragraph of this RESULTS section, ADP355 (355) inhibited K562 growth with maximum efficacy observed at 1 μM concentration. ADP400, Chex-DSer-8, counteracted ADP355 actions against K562 proliferation dose-dependently (Figure 3). However, as opposed to the 200 nM agonistic activity of ADP355, the IC_50_ of Chex-DSer-8 was just barely below 1 μM (920 nM) and the pharmacological profile did not reverse at high (in this case 10 μM) peptide concentrations.

#### Breast cancer model

ADP355 (peptide 355) attenuates MCF-7 BC cell growth by 20–25% when added at 100 nM concentration (Otvos et al., 2011a). In an experiment identical to that presented for K562 cells, Chex-DSer-8 (400) counteracted ADP355 cytostatic actions in MCF-7 cells in a dose-dependent manner with full activity observed at 100 nM concentration and an IC_50_ of approximately 15 nM (data not shown). This value is somewhat, but not significantly, higher than the IC_50_ figure of ADP355 itself against MCF-7 cells suggesting that in this concentration range Chex-DSer-8 (ADP400) is indeed an AdipoR antagonist and both peptides likely interact with the same receptor with similar avidity. The combination of the CML and BC findings suggests that modulation of AdipoR activity with our peptides is more pronounced for solid tumors than in hematologic cancers, and potentially reflecting low AdipoR levels in CML (Ozturk et al., 2012). Nevertheless, these data also show that peptide 400, Chex1-DSer-8, can be used with confidence as a sensitive AdipoR target validation tool in different biological models.

**Figure 4:**
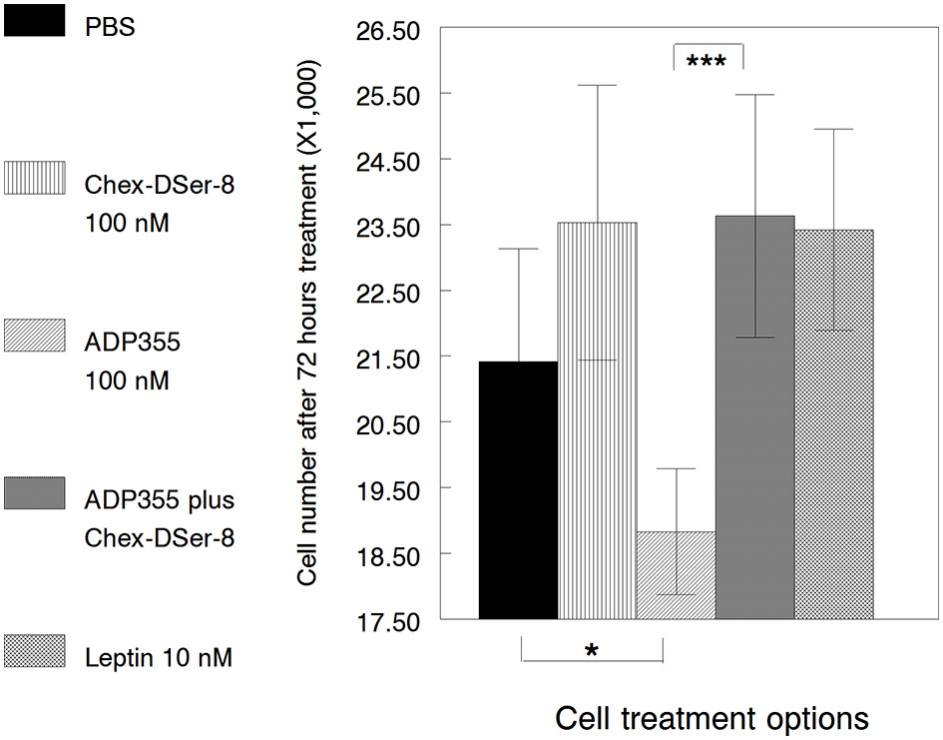
**Effects of adiponectin-derived peptides on the proliferation of MCF-7 cells**. The peptides (ADP355 and ADP400, Chex-DSer-8) were tested at 100 nM concentration. PBS indicates cell growth without any additives. Leptin protein added at 10 nM was used as a positive control for exogenous mitogen actions. Statistically significant differences (*p* ≤ 0.1 and 0.01) between samples are indicated by single and triple asterisks, respectively.

Up to this point we looked at the effects of the AdipoR antagonist ADP400, Chex-DSer-8, (ADP400) in the presence of agonist ADP355. However, MCF-7 cells express adiponectin themselves (Jarde et al., 2009), and it is possible that AdipoR antagonists promote cancer cell growth by interfering with autocrine adiponectin. It needs to be added that this hypothesis is untrue for our ObR antagonists that do not promote or inhibit MCF-7 cell growth when used alone (without leptin) in a wide concentration range (Otvos et al., 2011b,c). We repeated the MCF-7 growth promotion/inhibition assay with the adiponectin-derived peptides twice and noticed that on cell passage Chex-DSer-8 (peptide 400) at 100 nM concentration may have mitogenic properties. We incubated cells with leptin protein alone to quantify the additional level of cell proliferation, and indeed, 10 nM leptin promoted MCF-7 cell growth, as always, by 10% (Figure 4). Similar to earlier results, the AdipoR agonist ADP355 (355) at 100 nM attenuated MCF-7 cell growth with 90% statistical confidence (*p* = 0.092). Likewise, ADP400, Chex-DSer-8, at 100 nM completely ameliorated ADP355-induced growth inhibition (*p* = 0.003). However, in this instance cell proliferation was more extensive than without any peptide added and reached the extent of cell growth stimulated by the mitogen leptin. This effect was probably due to inhibition of the action of both exogenous and endogenous AdipoR agonists, as without ADP355 present, the AdipoR antagonist promoted cell growth to an identical degree.

### Molecular modeling of the native adiponectin active site, the ADP355 agonist and the ADP400 Chex-DSer-8 antagonist

**Figure 5:**
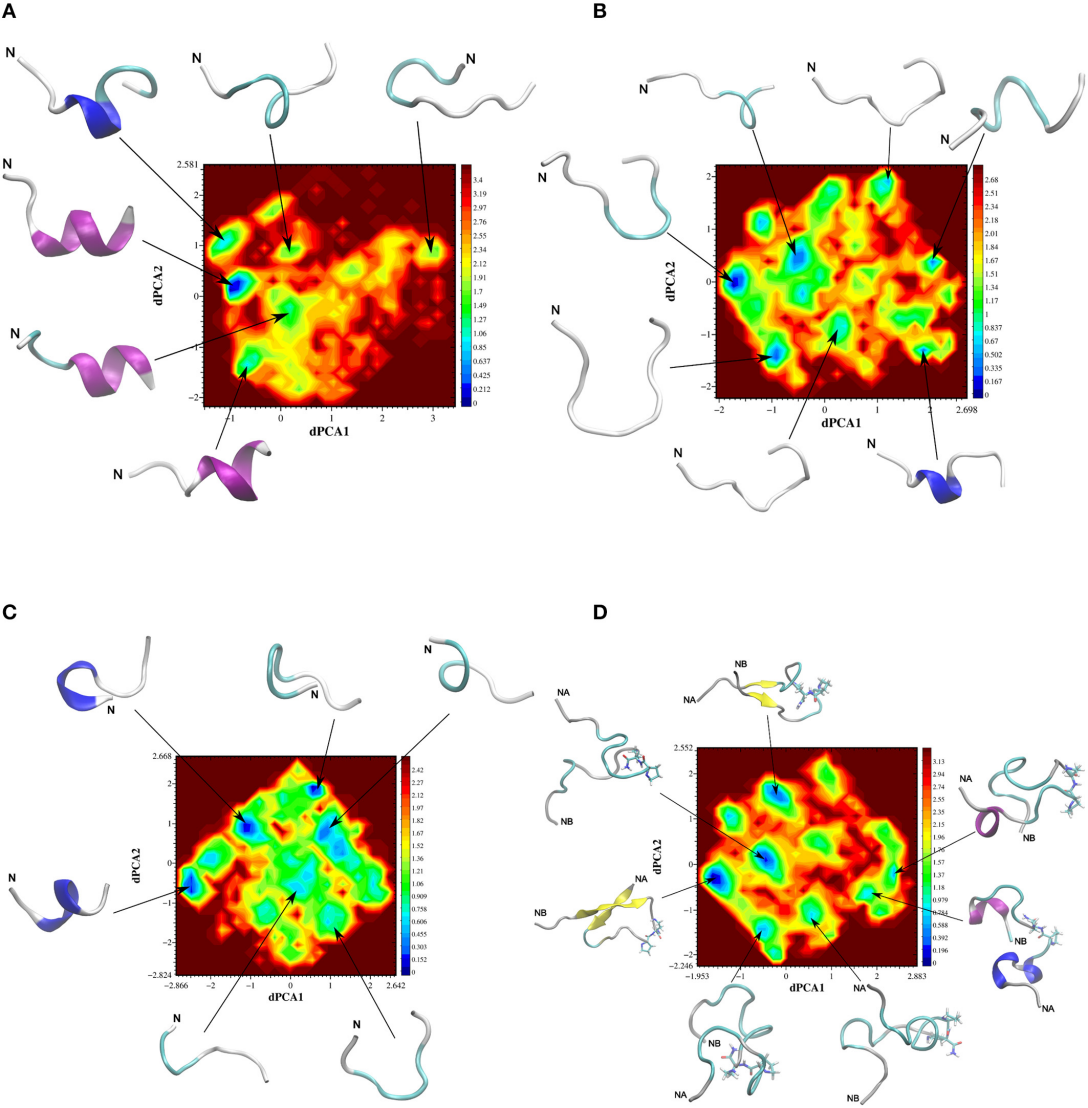
**Free energy surfaces (in kJ mol^−1^) of adiponectin peptide analogs as a function of the first two dihedral principal components**. The representative backbone structures located at different minima are indicated by an arrow, N indicates the N-terminus of a peptide chain, NA an NB on **(D)**, respectively, indicate the N-terminus of chains A and B. The corresponding color code for the free energy values are plotted as a bar at the right side of the surfaces. Color codes for secondary structures: pink, α-helix; blue, 3_10_-helix; yellow, β-sheet; cyan, β-bend; gray, random meander. **(A)** Free energy surfaces (in kJ mol^−1^) of native peptide 25; **(B)** free energy surfaces (in kJ mol^−1^) of first generation agonist peptide ADP355 (355); **(C)** free energy surfaces (in kJ mol^−1^) of antagonist peptide 400; **(D)** free energy surfaces (in kJ mol^−1^) of linear branched dimeric peptide 399.

**Figure 6:**
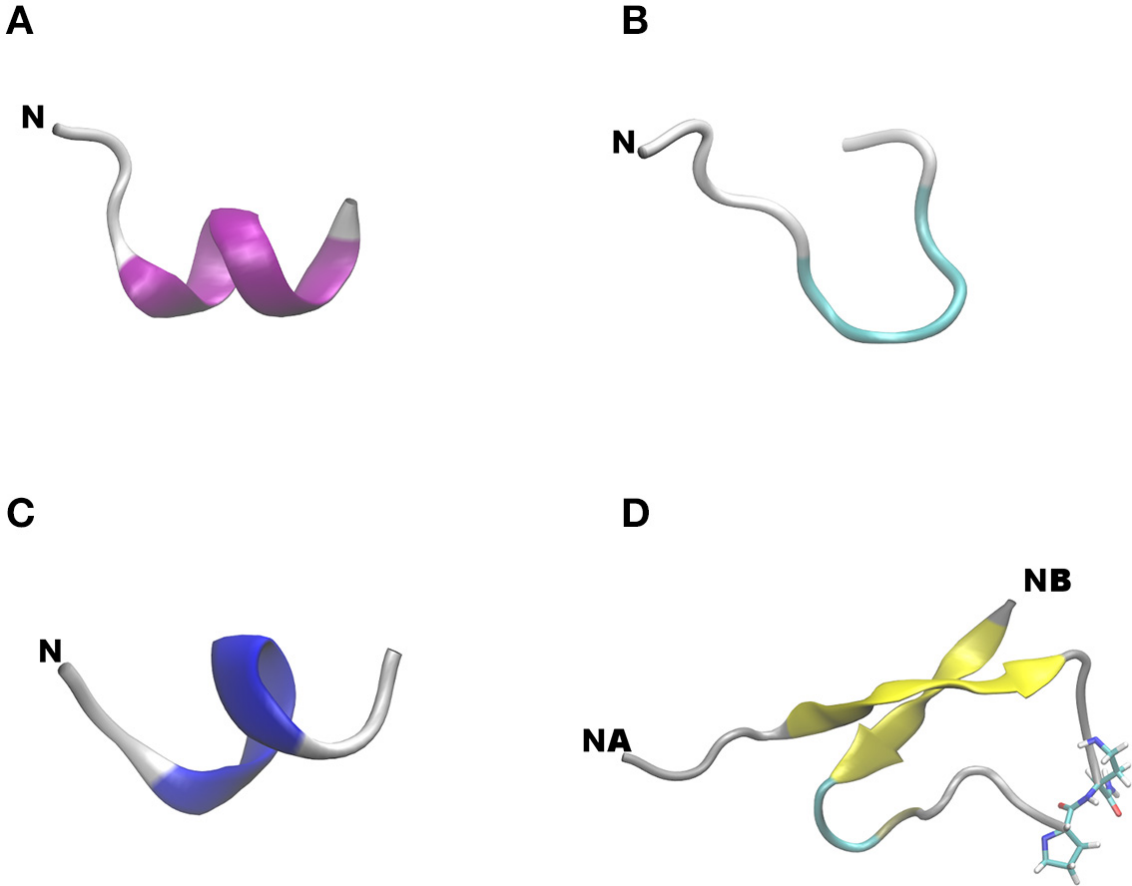
**Lowest energy backbone structure of peptides**. **(A)** Native peptide 25; **(B)** monomeric agonist peptide 355; **(C)** antagonist peptide 400; **(D)** branched dimeric peptide 399. N indicates the N-terminus of a peptide chain, NA, and NB on **(D)**, respectively, indicate the N-terminus of chains A and B. Color codes for secondary structures: pink, α-helix; blue, 3_10_-helix; yellow, β-sheet; cyan, β-bend; gray, random meander.

Dihedral principal component analysis of the last 50 ns of the replica-exchange molecular dynamics (REMD) trajectory revealed that the free energy landscape has multiple minima with one deep local minimum (Figure 5). The lowest energy structures of the native adiponectin active site (peptide 25, Ile-Pro-Gly-Leu-Tyr-Tyr-Phe-Ala, Table 2) assumed a mixture of random and α-helical conformations in the global minimum and were in predominantly α-helices in the rest of the low energy structures (Figure 5). Turn conformations were also located in higher energy local minima, but these occupied shallow configurational space. Taken together the helical structure seemed to be quite stable. In contrast, on the free energy surface of ADP355 more minima were observed (Figure 5). The free energy surface was shallower than that of peptide 25 indicating remarkable flexibility of the peptide. In the global minimum (Figure 6) a turn conformation was present in the middle of the peptide (resembling a β-hairpin conformation). The other structures were mostly random in the higher energy minima. Apparently, the designer peptidomimetic ADP355 was considerably more flexible than the native fragment, and such could fit into the AdipoR groove with smaller energy expense than the unmodified parent peptide.

The free energy surface for peptide 400 (Chex-DSer-8 antagonist) was even more shallow than the previous two surfaces indicating a flexible structure, but a central 3_10_-helix conformation dominated the global minimum (Figure 5). When the helix was not visible, turns populated the lower energy conformers. These structural features allowed the peptide to assume the required receptor-bound conformation and antagonize adiponectin actions. The somewhat increased flexibility of ADP355 may explain the slightly improved binding constants relative to Chex-DSer-8 (ADP400).

Based on the present REMD data, three novel AdipoR agonists were designed (Table 1 and Discussion). Two of these were dimeric structures with one of them having a head-to-tail cyclic structure (peptide 398), and the other being a linear branched dimer C-terminally tethered to a diamino-butyric acid scaffold (peptide 399). The third peptide was a trimer of ADP355 (ADP500, 500). The free energy surface of the linear dimeric peptide 399 using the first two principal components, dPCA1 and dPCA2, of chain A were similar to those of the other three peptides and exhibited multiple minima (Figure 5). However, here the lowest energy structure was in a deep well and the two peptide chains were in a twisted anti-parallel β-sheet conformation (Figure 6). The β-sheet conformation was also a feature of the third lowest energy structure, however, here the two chains were in parallel orientation. In other minima the individual chains showed similar structural features to the single chain peptide 355.

### Activity of the second generation AdipoR agonists

The activity of the dimeric and trimeric peptides 398, 399, and 500 was tested in both cancer cell lines of this study. First the K562 CML cell line was used that is less responsive to ADP355 (355) treatment than MCF-7 BC cells and thus the efficacy differences among analogs were expected to be easier to observe. The cyclic dimer 398 inhibited K562 proliferation to a similar extent as the parent ADP355 analog (Figure 1). Maximal efficacy was detected at 1 μM peptide concentration with a relative IC_50_ of 300 nM. In contrast, the linear branched dimer (ADP399) exhibited significantly improved activities, with maximal effects at 100 nM and IC_50_ value of 10 nM (Figure 2). Similar to the observations regarding the parent peptide ADP355, in later assays/cell passage the maximum activity of ADP399 was improved, and was as low as 10 nM. The 20-fold increase (molar terms, 8-fold increase in w/v terms) in the activity upon lead optimization is strikingly similar to the improvement in cancer cell proliferation inhibition when going from first to second generation ObR antagonists (Otvos et al., 2011b,c). As not only the concentration-activity curve shapes were identical but all three AdipoR agonist peptides (having identical base sequences) restricted tumor cell growth to an identical degree (in the presented assay at 40%) at their maximal activity concentration, we assume that the mode of action of ADP355, the cyclic dimer and the linear dimer are also identical. The branched trimer (ADP500) was inactive against K562 cells up to 1 μM concentration.

For MCF-7 cells, significant (>15%) growth inhibition was observed for ADP355 at 10 nM and the linear dimer between 100 pM and 10 nM concentrations (Table 3). The approximately 10-fold improved activity of ADP355 compared to our earlier report (Otvos et al., 2011a) and Figure 4 of this account is likely due to slightly different experimental conditions. In this measure, the cyclic dimer appeared to be less efficacious against MCF-7 cell proliferation than the original peptide ADP355. Nevertheless, against MCF-7 cells, similar to K562, the linear dimer was by magnitudes more active than any of the other two peptides (Table 3). Addition of the third ADP355 chain in peptide ADP500 (500) did not provide any growth inhibitory advantage over the linear dimer at the 100 pM–10 nM concentration range. In fact at 10 nM concentration, the activity was found similar to the base peptide ADP355. Due to the significantly increased synthetic difficulties and costs of ADP500 compared to ADP355 and ADP399, the trimer was no longer studied.

**Table 3:** **Inhibition (percent of untreated control) of MCF-7 BC cell proliferation by adiponectin active site analogs**.

**Peptide concentration**	**ADP355 (355)**	**Cyclic dimer (398)**	**Linear dimer (399)**
10 pM	−5	13	−4
100 pM	12	4	***27***
1 nM	8	7	14
10 nM	*17*	5	*16*
100 nM	2	−1	1

*The table shows the mean of two independent experiments, each ran in one plate, in duplicates with multiple readings. Very significant (>25%, marked in bold and italics) and significant (>15%, marked in italics) growth inhibition was observed for ADP355 at 10 nM and the linear dimer between 100 pM and 10 nM concentrations*.

Similar to the neutralization of the growth restricting effects of ADP355, the AdipoR antagonist Chex-DSer-8 (400) fully counteracted the activity of the linear dimer (399). During yet another repetition of this 3-day growth inhibition assay, at 10 nM concentration, the linear dimer reduced the number of viable MCF-7 BC cells by 25%, but when the cells were co-treated with the linear dimer (ADP399) and the Chex-D-Ser-8 (ADP400) (both at 10 nM), the number of cells was practically identical to that in untreated cultures (1% reduction). This data indicates that (i) the linear dimer and Chex-DSer-8 interact with the same receptor, and (ii) that the antagonist peptide (400) does not exert pro-mitogenic properties at lower concentrations.

### Serum stability of AdipoR agonists

Peptide ADP355 (355) is remarkably stable in mouse serum (Otvos et al., 2011a). Based on the degradation kinetics in diluted serum, the half-life in whole plasma is estimated at 75 min. To assess the pharmaceutical utility of the new adiponectin peptides here we compared the degradation kinetics of two AdipoR agonists in undiluted mouse serum. For ADP355, experimental data provided proof for even more extensive protease resistance than the calculations suggested, exhibiting a serum half-life of 180 min (data not shown). This stability level is quite high among peptide therapeutics, and is likely due to the incorporation of 4 unnatural amino acid moieties, including three D-residues that significantly improve peptide stability (Powell et al., 1993). The linear branched dimeric construct 399 was somewhat less stable indicating that the modifications aimed at increasing the activity reduced the resistance to proteolytic degradation but in general kept the analog among the most stable peptide drug leads. The half-life of the linear dimer ADP399 was 55 min with 16% intact peptide still present after 2 h incubation with mouse serum. The cyclic dimer precipitated during the assay procedure, and thus its quantities could not be accurately measured.

### Biodistribution

**Figure 7:**
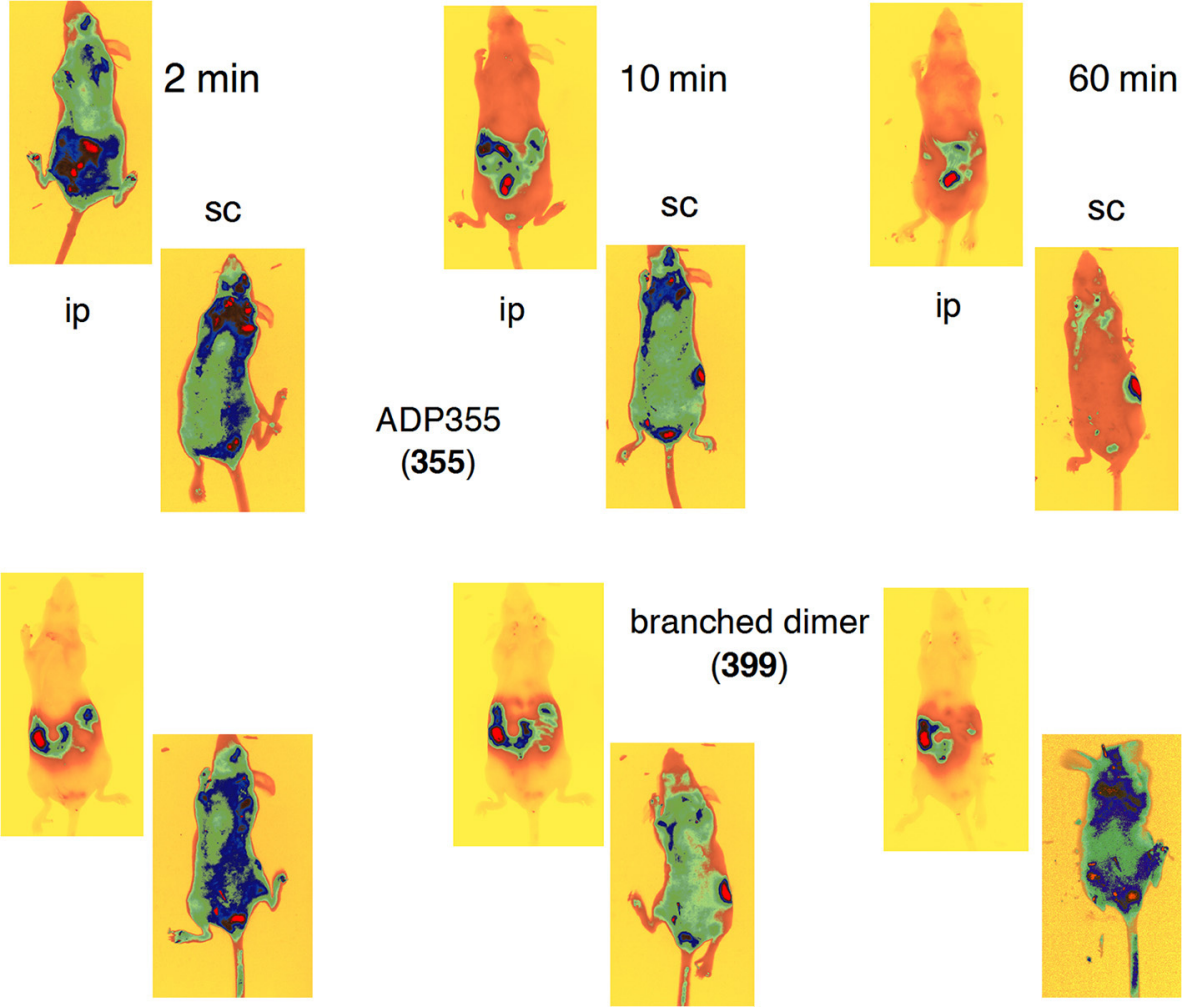
**Biodistribution of N-terminally DY675-labeled peptides in Skh1 hairless mice**. The top two rows show the absorption of peptide ADP355 (355) and the bottom two rows of that of the linear dimer (399). The first and third rows represent intraperitoneal (ip), the second and fourth document subcutaneous (sc) peptide administration.

Immediately (1 min handling +1 min exposure) upon ip administration to an Skh1 mouse, N-terminally DY675-labeled peptide ADP355 (355) was distributed into the usual peptide elimination organs (Klootwijk et al., 1997): the kidneys, the urinary bladder, the liver, and the rectum (Figure 7). In addition, the labeled peptide migrated into all tissues, albeit less efficaciously. Apparently ADP355 was quickly eliminated from the circulation as at 10 min and beyond it was observable only in the kidney and the bladder (Figure 7). Importantly, at the earliest timepoint ADP355 was observed in the brain area, similarly to our ObR response modifier peptides (Otvos et al., 2011b; Beccari et al., 2013). While in all these assays it cannot be excluded that cleaved fluorophore skews data interpretation, the leptin peptides and an antimicrobial peptide labeled in an identical manner display clearly different biodistribution patterns (Otvos et al., 2008; Rozgonyi et al., 2009). The branched linear dimer peptide 399 was distributed less actively and remained in the peptide elimination organs throughout the examination procedure (Figure 7). The increased size of the dimer compared to the monomer may explain the reduced ability to enter tissues.

A markedly different picture emerged when the two peptides were administered sc. Peptide ADP355 (355) was clearly better absorbed (compared to ip administration) because strong and homogenous biodistribution could be observed even at the 10 min time point (Figure 7). Remarkably, upon sc administration the branched linear dimer was retained throughout the body in the entire 60-min examination period (Figure 7). Although at the hour time point significant amounts of peptides were already migrated to the kidneys and the bladder, still strong remaining staining was observed in the lungs, upper abdomen, and the brain. Judged from the biodistribution pattern, the most promising analog for *in vivo* efficacy studies is the branched linear dimer, administered sc.

## Discussion

When removed from the protein environment, earlier molecular dynamics studies indicate that the isolated native adiponectin active site loses the β-pleated sheet character it adopts in the protein and forms a series of turns (Otvos et al., 2011a). The designer peptidomimetic, ADP355 appears to fold into a more stable conformation characterized by a hairpin incorporating almost the entire peptide. The current molecular modeling utilized the state-of-the-art REMD simulation protocol with the AMBER99SB-ILDN-NMR force field (Li and Bruschweiler, 2010). When this force field, together with many other modern force fields, was tested it achieved the highest accuracy in extensive benchmark calculations (Beauchamp et al., 2012). The present calculations agree with the previous one in the higher conformational freedom of ADP355 compared to the native sequence with a turn in the middle of the peptidomimetic. The only difference between the results generated by the two methods is the increased α-helix and decreased β-sheet contribution to the structure of the native active site after REMD simulations.

These MD calculations served as base for the design of second generation AdipoR agonist peptides. As adiponectin circulates in higher order complexes, we wanted to develop a dimer and a trimer of the first-in-class AdipoR agonist peptide ADP355. Higher order multimers were not considered due to anticipated synthetic difficulties and avoiding potential aggregation of the identical chains that could be even further strengthened by parallel peptide segments induced by the central hairpin. To show the importance of the conformational freedom in binding AdipoR, we designed a linear branched peptide that was expected to exhibit stronger activity due to the multimeric chain arrangement. To make the ADP355 fragment freely available for receptor binding in the dimeric construct, we C-terminally extended the monomer sequence by His, the amino acid residue following the native Tyr in the active site and a turn-forming Pro residue. The peptide retained conformational flexibility but the lowest energy structure is in an anti-parallel β-sheet conformation, which is most likely preferred by the receptor. The dimer was designed on a template from the native adiponectin active site (peptide 25) which is in the adiponectin protein in an anti-parallel β-sheet conformation (Otvos et al., 2011a). Subsequently, the improved receptor affinity of the dimer is most likely due to mimicking the native conformation of adiponectin. We also designed the same peptide embedded into a cyclic structure. The cyclic peptide has less conformational freedom and such was expected to exhibit weaker cellular activities in spite of the multicopy agonist arrangement. In both new designs we extended the sequence with a C-terminal His (the native residue following Tyr that is mimicked by DSer10 in ADP355) and turn-forming Pro and Asp residues to separate the individual chains within the same peptide. As the trimer exhibited no cellular therapeutic advantage over the dimer, its conformation was not studied in detail.

The cellular models used here were selected among those known to express AdipoR, involve adiponectin in biological responses and yield reproducible results in our hands. Adiponectin protein has been linked to leukemia, lymphoma, and myeloma in individuals with above-average BMI (Obeid and Hebbard, 2012). Human-origin K562 CML cells express both AdipoR forms although at relatively low levels (Ozturk et al., 2012). Imatinib chemotherapy does not affect AdipoR expression in K562 cells, suggesting a potential combination treatment strategy with BCR-ABL tyrosine kinase inhibitors and AdipoR agonists. In the earliest report on the subject, addition of adiponectin protein inhibits the relative proliferation rate (compared to the unrelated human serum albumin) of K562 cells by 9% (Yokota et al., 2000). The higher level of activity of our peptidic adiponectin fragments on K562 proliferation reflects the previously observed increased activity of our agonist using glioma and BC cells compared to the full protein (Otvos et al., 2011a). Alternatively, processed fragments of adiponectin exhibit improved cellular activities (*vide supra*). While the role of adiponectin in kidney fibrosis and myofibroblast differentiation is controversial (Beige et al., 2009; Yang et al., 2013) the adipokine appears to prevent interstitial fibrosis *in vivo* (Rutkowski et al., 2013) and fibroblast migration and transition to myofibroblasts *in vitro* (Cai et al., 2010). Here we tested whether peptide ADP355 can ameliorate fibrosis by interfering with differentiation to myofibroblasts, a marker of activation in fibroblasts. Previous studies suggest that the herein used renal interstitial fibroblasts isolated from fibrotic rat kidneys are more active in terms of proliferation and synthetic activity than fibroblasts grown from normal kidney (Rodemann and Muller, 1990) and are therefore a better reflection of their *in vivo* counterparts. Importantly, we wanted to compare the cellular IC_50_ values of ADP355 across divergent diseases to obtain a uniform baseline during the improvement of the activity figures.

The assay repertoire for evaluating the relative efficacy of adiponectin-based peptides is very limited. Conceivably, short adiponectin fragments, such as our peptides, further expand the portfolio of biological responses in adiponectin-sensitive cells. Thus, with no reliable external control available (including endogenously induced or exogenously added full-sized adiponectin protein), we had to compare the activity of the new derivatives to the base peptide ADP355 (355), very heavily studied for its *in vitro* and *in vivo* functions earlier (Otvos et al., 2011a; Pepping et al., 2014), in cancer cell lines whose easily measurable proliferation properties were repeatedly shown to be characteristic to typical adiponectin functions (Jarde et al., 2009; Ozturk et al., 2012). The cellular models meant to investigate adiponectin receptor-mediated biological functions. Originally we used siRNA knockdown to show that ADP355 affects AdipoR responses, especially those proceeding through AdipoR1 activation (Otvos et al., 2011a). Because the peptide analogs studied in this report have almost identical sequences as ADP355 just differ in size or valency, we can safely assume that these also influence AdipoR signaling. With all the cell line and assay condition dependence of signature AdipoR cellular responses (Wijesekara et al., 2010; Otvos et al., 2011a) it is very difficult to identify and test characteristic signaling events. Moreover, in our hands, as well as in the literature (cf INTRODUCTION), various adiponectin protein preparations induce diverse cellular responses, making almost impossible to identify a standard assay that would prove AdipoR activation or deactivation. The most straightforward measure of peptide-receptor interactions would be the studying of direct molecular binding. We attempted to accomplish that with DY675-labeled ADP355 (355) and linear dimer (399) and a commercially available AdipoR1 preparation (Novus Biochemicals) using both enzyme-linked immunosorbent (ELISA) and dot-bot type assays. Due to the notorious insolubility of AdipoR we immobilized it to the solid surfaces and to the reduction of the binding efficacy when labels are near to adipokine active sites (Otvos et al., 2014) we focused on 10 nM—1 μM soluble labeled peptides. Unfortunately while at 100 nM we detected very low level of specific binding (more for ADP399 than for ADP355), at 1 μM both DY675 conjugated peptides adhered to the plastic and paper surfaces in spite of previous blocking of these sites and extensive washing with aqueous buffers.

The ADP399 linear dimer seems to fulfill the criteria of contemporary drug leads. The 10 nM relative IC_50_ value is quite promising in CML therapy (or in fact any other diseases where the adiponectin receptor needs activation) on its own right. Significantly, the improvement in efficacy was not accompanied with major loss of metabolic stability. Although ADP355 is more stable in serum than the linear dimer, the serum metabolites of the dimer may also include active fragments. A preliminary mass spectrometry study of the serum metabolites of the linear branched dimer peptide 399 suggested two degradation pathways. In one of them one of the chains remains intact, while in the other chain the N-terminal five (2233 Da) and six (2069 Da) residues are cleaved. Thus, a complete chain, with full AdipoR agonist properties is still present among the degradation products. This is very similar how the antibacterial peptide A3-APO dimer, which is similarly built on a C-terminal Dab scaffold, breaks down *in vivo* at the tethering point into two complete peptide chains that retain full *in vitro* and *in vivo* activities (Noto et al., 2008).

Why the ADP399 linear branched dimer (399) is more active than the monomer 355 (ADP355) needs further experimentation. We designed the dimers because adiponectin protein circulates in trimeric and higher order complexes. The dimeric structure seems to induce an antiparallel β-pleated sheet structure (Figure 6D) but under our experimental conditions it did not readily form aggregates. Furthermore, peptide dimers are generally more active than single chain analogs, even in applications when the native parent peptide is monomeric (Cudic et al., 2002; Janssen et al., 2002; Aggarwal et al., 2006). Potentially simultaneous inhibition of multiple receptor copies, or in general terms interaction with multiple copies of cell surface structures, offers a therapeutic advantage for peptide drugs. MD calculations suggested that the charge density-based binding affinity, stable covalent dimerization, and the ability to dimerize and adopt a well-defined structure are important physicochemical properties of a C-terminally tethered antibacterial peptide that exhibits improved activity relative to the monomeric version (Zhou et al., 2011). The cyclic AdipoR agonist dimer 398 did aggregate during the serum stability assay, but we did not identify the exact step when this aggregation occurred. The slight loss of activity compared to ADP355 could be due to the restriction in conformational freedom, to aggregation, or both. The trimeric variant (500) had an activity profile similar to those of other peptides in this study against MCF-7 cells without full inhibition by the AdipoR antagonist (400) suggesting potentially additional targets.

Many peptide drugs, including our antibacterial peptides, demonstrate inferior *in vivo* efficacy when added sc compared to ip administration and we explained this with the poor drug absorption during the sc route (i.e., the peptide remains at the injection site as a depot) (Rozgonyi et al., 2009). Notable exceptions to this rule are insulin that is administered at home sc, and our leptin receptor response modifier peptide derivatives (Knappe et al., 2008; Beccari et al., 2013). In fact, our leptin receptor antagonist Allo-aca is more efficacious in animal models of BC when added sc than when administered ip (Otvos et al., 2011b). The explanation of the improved biodistribution of adipokine analogs lies in the very extensive vascularization of the subcutaneous adipose tissue (Gaelekman et al., 2011). Capillary density per adipocyte is higher in subcutaneous than in visceral tissue offering fast and widespread absorption into the systemic circulation upon sc drug administration. In addition, overexpression of adiponectin in mice was shown to result in highly vascularized subcutaneous adipose tissue (Rutkowski et al., 2009), further improving the potential success of adiponectin-based drugs upon sc inoculation.

This is a first report on the development on an AdipoR antagonist peptide. Due to the high sequence similarities peptide ADP400, Chex-DSer-8 (400), is almost as efficacious an antagonist as the parent ADP355 (355) is an agonist. Because adiponectin restricts MCF-7 cell proliferation, these cells express just small amounts of the adipokine (Jarde et al., 2009). At the lowest concentration that elicits maximal activity (100 nM) the Chex-DSer-8 produces minor mitogenic response in MCF-7 cells (depending upon the origin of the test cell population), perhaps by antagonizing the autocrine adiponectin loop. Alternatively, ADP400, Chex-DSer-8, can act as an inverse AdipoR agonist. An analogy with ObR antagonist peptides may justify this assumption. In most tumor cells, the ObR antagonist Allo-aca exhibits a large concentration window (>1000-fold) from going from antagonist to agonist (Otvos et al., 2011b,c). Against the K562 CML cell line which expresses low levels of ObR (Ozturk et al., 2012), however, Allo-aca either retains anti-proliferative activity without exogenous leptin added, or exhibits a narrower concentration window (approximately 100-fold) before it stimulates rather than inhibits cell growth depending upon the nutrient strength of the media or the cell passage (*vide supra*) (Otvos et al., 2014). Apparently at low receptor expression levels adipokine receptor antagonists may exhibit inverse agonist properties. In any event, with submicromolar activities, peptide Chex-DSer-8 should be able to perform well all across medicine as an AdipoR target validation tool. As a therapy agent, for treating arthritic diseases lead optimization should be able to improve the activity 10–50 fold similar to our second generation AdipoR agonists reported herein, as well as optimized ObR agonist and antagonist peptides we developed earlier (Kovalszky et al., 2010; Otvos et al., 2011b). A remote possibility for the therapeutic use of an AdipoR antagonist is type 1 diabetes, a condition in which elevated levels of both the receptor and the ligand were found in patients and disease-carrying experimental animals (Karamifar et al., 2013; Lin et al., 2013).

## Author contributions

Laszlo Otvos, Eva Surmacz, assay design, data analysis, manuscript preparation; Daniel Knappe, Ralf Hoffmann, Feng Lin, John D. Wade, peptide synthesis and analysis, serum stability, manuscript preparation; Ilona Kovalszky, Julia Olah, cellular studies, and biodistribution; Tim D. Hewitson, myofibroblast experiments; Roma Stawikowska, Maciej Stawikowski, Predrag Cudic, receptor binding; Hungarian letter order of Sandor Lovas, molecular modeling, manuscript preparation.

### Conflict of interest statement

The authors declare that the research was conducted in the absence of any commercial or financial relationships that could be construed as a potential conflict of interest.

## Abbreviations

AdipoR: adiponectin receptor
All: allyl
ADP355: first generation adiponectin receptor peptide agonist
ADP399: second generation adiponectin receptor agonist, linear dimer of ADP355
ADP400: adiponectin receptor antagonist/inverse agonist
AMPK: 5′ adenosine monophosphate-activated protein kinase
BC: breast cancer
BMI: body mass index
BSA: bovine serum albumin
Chex: 1-amino-cyclohexane carboxylic acid
CML: chronic myeloid leukemia
Dab: 2,3-diamino butyric acid
DIC: diisopropyl-carbodiimide
dPCA: dihedral principal component analysis
DMEM: Dulbecco's modified Eagle's medium
DMF: dimethyl formamide
EC: effective concentration
ELISA: enzyme-linked immunosorbent assay
FBS: fetal bovine serum
Fmoc: 9-fluorenyl-methoxycarbonyl
GLP: glucagon-like peptide
HOBt: 1-hydroxy-benzotriazole
IC: inhibitory concentration
ip: intraperitoneally
MALDI TOF-MS: matrix-assisted laser ionization/desorption time-of-flight mass spectrometry
MBHA: 4 methylbenzhydrylamine
ObR: leptin receptor
PBS: phosphate buffered saline
REMD: replica-exchange molecular dynamics
RP-HPLC: reversed-phase high performance liquid chromatography
SMA: α-smooth muscle actin
sc: subcutaneously
SE: standard error
TFA: trifluoroacetic acid.
